# Supplementary material for: The Systems Biology Research Tool: evolvable open-source software
Source: BMC Syst Biol. 2008 Jun 29;2:55. doi: 10.1186/1752-0509-2-55 (PMC2446383; doi:10.1186/1752-0509-2-55)
Supplement: Additional file 1 — SBRT Archive. An archive of the current version of the Systems Biology Research Tool. [file 1752-0509-2-55-S1.zip › sbrt-1.4.0/doc/developers_guide/api/allclasses-noframe.html]

All Classes


**All Classes**
  

AbstractCvComputer
  
AbstractFluxIntervalComputer
  
AbstractLinearComb
  
AbstractLinearComb.Term
  
AbstractLinkedHashSetFormat
  
AbstractListFormat
  
AbstractMapEntryFormat
  
AbstractMapFormat
  
AbstractMapInputFile
  
AbstractMapOutputFile
  
AbstractMdMatrix
  
AbstractPolynomial
  
AbstractProcessManager
  
AbstractProgressMonitor
  
AbstractResultsManager
  
AbstractRxn
  
AbstractSbrtFile
  
AbstractSmdMatrix
  
AmpersandSetFormat
  
And
  
*AppInputFileLineFormat*
  
AppInputFileLineFormatVersion1
  
ApplicationException
  
BiggBracketParserV1
  
BiggRxnNodeParser
  
BiggSbmlReader
  
BiggSbmlReaderManager
  
BiggSbmlTranslater
  
BiggSbmlTranslaterManager
  
*BooleanFormat*
  
BooleanRelation
  
BoolRelFormatV1
  
BoundedIntervalFormatV1
  
CatalystDeletionManager
  
CatalystDeletionProcess
  
*CatalystFormat*
  
CatalystFormatV1
  
CatalystFormatV2
  
*CatalystListFormat*
  
CatalystListFormatV1
  
*CatalystSetFormat*
  
CatalystSetFormatV1
  
CatalystVerifier
  
*CatalyzedFluxome*
  
CdHarComputer
  
CdHarFluxVectorComputer
  
CdHarFluxVectorManager
  
CdHarFluxVectorProcess
  
CdHarManager
  
CdHarProcess
  
*ChemRxn*
  
*ChemSpeciesFormat*
  
ChemSpeciesFormatV1
  
ChemSpeciesVerifier
  
*CoeffFormat*
  
CoeffFormatV1
  
CoeffFormatV2
  
CofoofSource
  
*CollectionFormat*
  
ColonMapEntryFormatV1
  
ColonMapFormatV1
  
ColumnOrFbaOptOutputFile
  
ComiFile
  
ComiFileSource
  
ComoFile
  
ComoFileSource
  
ConflictingInputException
  
*ConstraintsFileLineFormat*
  
ConstraintsFileLineFormatV1
  
ConstraintsFileLineFormatV2
  
ConstraintViolationException
  
*ConvexPolytope*
  
CorrelationAnalysis
  
CorrelationManager
  
*Correlator*
  
CorrResFormatterV1
  
*CorrResult*
  
*CorrResultsFormatter*
  
CplexSolver
  
CvCatalystDeletionManager
  
CvCatalystDeletionProcess
  
CvFbaOptimizationExperiment
  
CvFbaOptManager
  
CvFbaOptProcess
  
CvFluxIntervalProcess
  
CvMsFluxIntervalManager
  
CvObjectiveFunctionManager
  
CvObjectiveFunctionProcess
  
CvRevRxnFluxIntervalManager
  
CvRxnDeletionManager
  
CvRxnDeletionProcess
  
CvSimpleFluxIntervalManager
  
*CycleComputer*
  
CycleIdManager
  
CycleIdProcess
  
CycleIdProcess
  
DevNull
  
DimensionException
  
DirectedGraph
  
DirectedGraph.Edge
  
*DirEdgeFormat*
  
DirEdgeFormatV1
  
DiscreteProgressMonitor
  
DoubleEquiv
  
*DoubleFormat*
  
DoubleFormatV1
  
DoubleFormatV2
  
*DoubleMatrix*
  
*DoubleMatrixBuilder*
  
DoubleMatrixWrap
  
DummyConsole
  
EndOfFile
  
EqualsMapEntryFormatV1
  
EqualsMapFormatV1
  
*EquivTester*
  
EuclideanDist
  
ExceededLimitException
  
ExchangeRxn
  
ExternalSoftwareException
  
*ExtremeCurrentComputer*
  
ExtremeCurrentManager
  
ExtremeCurrentManager.Rm
  
ExtremeCurrentProcess
  
FastMsFluxRangeComputer
  
FatalException
  
FbaManager
  
FbaOptComputer
  
FbaOptException
  
*FbaOptFileLineFormat*
  
FbaOptFileLineFormatV1
  
*FbaOptHeaderFormatter*
  
FbaOptHeaderFormatterV1
  
*FbaOptHeaderParser*
  
FbaOptHeaderParserV1
  
FbaOptimizationProcess
  
FbaOptManager
  
*FbaOptOutputFile*
  
FbaProcess
  
FbaSolver
  
FbaSolverManager
  
FbaSolverProcess
  
FileFormat
  
FileFormatFormat
  
FileLineIterator
  
FluxActivityManager
  
FluxActivityProcess
  
FluxActivityProcess.Results
  
FluxCapComputer
  
FluxCapException
  
*FluxCapFormat*
  
FluxCapFormatV1
  
FluxCapInputFile
  
FluxCapManager
  
FluxCapProcess
  
FluxConstraints
  
*FluxCvComputer*
  
FluxIntervalAnalyzer
  
*FluxIntervalComputer*
  
FluxIntervalInputFile
  
FluxIntervalProcess
  
*Fluxome*
  
*FluxomeReducer*
  
FluxomeReducerProcess
  
FluxomeSolFileLineFormatV1
  
*FluxomeSolutionFileLineFormat*
  
FluxPlasticityManager
  
FluxPlasticityProcess
  
FluxPlasticityProcess.Results
  
FluxRangeException
  
*FluxVectorFormat*
  
FluxVectorFormatV1
  
*Format*
  
FormatException
  
*Formatter*
  
GlpkSolver
  
Hamming
  
HashConvexPolytope
  
HashConvexPolytopeOld
  
HashLinearComb
  
HashLinearComb.Term
  
HashLinearSystemSolution
  
HashMatrix
  
IllegalBoundsException
  
IllegalFreeVariableException
  
IndexPair
  
*IndexPairFormat*
  
IndexPairFormatV1
  
InfeasibleSolutionException
  
InitialPointComputer
  
InitialPointGenerator
  
InitialPointManager
  
*InputFile*
  
*IntegerFormat*
  
IntegerFormatV1
  
Interval
  
IntervalComparer
  
IntervalComparer.Diff
  
IntervalComparison
  
IntervalComparisonManager
  
IntervalEquiv
  
*IntervalFormat*
  
IntervalFormatV1
  
IntervalVariationManager
  
InvalidFluxVectorException
  
InvalidPointException
  
IrfFormatV1
  
IrrevRxn
  
*IrrevRxnFluxome*
  
IrrevRxnFluxomeV1
  
IrrevRxnFormatV1
  
IrrevRxnFormatV1
  
IrrevRxnFormatV2
  
JriException
  
KendallTau
  
KendallTauA
  
KendallTauB
  
KendallTauManager
  
*KernelProcess*
  
*KernelProcessManager*
  
LinCombTermFormatV1
  
LinCombTermFormatV2
  
*LinearComb*
  
*LinearComb.Term*
  
LinearCombConverterManager
  
*LinearCombFormat*
  
LinearCombFormatV1
  
LinearCombFormatV2
  
*LinearCombTermFormat*
  
*LinearProgramSolver*
  
LinearSolverManager
  
LinearSolverProcess
  
*LinearSystemSolution*
  
*LinearSystemSolver*
  
*LinkedHashSetFormat*
  
*ListFormat*
  
ListResultsManager
  
*LongFormat*
  
LongFormatV1
  
*ManagerInput*
  
MannWhitneyResults
  
MannWhitneyU
  
MannWhitneyUManager
  
*MapEntryFormat*
  
*MapFormat*
  
*MapInputFile*
  
MapManagerInput
  
*MapOutputFile*
  
*MapTextFile*
  
*MapTextInputFile*
  
*MapTextOutputFile*
  
MathematicaException
  
MathematicaSolver
  
*MathExpr*
  
*MathExprFormat*
  
*Matrix*
  
*MatrixBuilder*
  
MatrixConverterManager
  
MatrixElement
  
*MatrixElementFormat*
  
MatrixElementFormatV1
  
MatrixMultiplier
  
MatrixTransposer
  
MatrixWrap
  
MetatoolFileWriter
  
MetatoolFileWriterManager
  
MissingVariableException
  
MMaExprInterpretter
  
MmaNotebookManager
  
*MonitoredProcess*
  
MsFluxIntervalComputer
  
MsFluxIntervalManager
  
MsFluxomeReducer
  
MsFluxomeReducerManager
  
MultiOutputFiles
  
MultipleCorrelationAnalysis
  
MultipleVectorsConverterManager
  
*MutableProgressMonitor*
  
MwUAnalysis
  
NetworkInfoManager
  
NetworkInfoProcess
  
NetworkInfoProcess.Results
  
NonEmptyStringFormat
  
NormalDist
  
ObjectiveFunctionAnalysis
  
ObjectiveFunctionManager
  
OptimizationSense
  
Or
  
*OutputFile*
  
PalssonRxnNodeParser
  
PalssonSbmlReader
  
PalssonSbmlReaderManager
  
PalssonSbmlTranslater
  
PalssonSbmlTranslaterManager
  
ParallelHyperplanes
  
*Parser*
  
Path
  
PathFinder
  
PathIdManager
  
PathIdProcess
  
*PercentFormat*
  
PercentFormatVersion1
  
PercentProgressMonitor
  
PipeListFormat
  
PipeSetFormat
  
*Polynomial*
  
*Polynomial.Term*
  
ProcessInputFile
  
*ProcessManager*
  
ProcessManagerFactory
  
ProcessRunner
  
*ProgramSolver*
  
ProgramSolverException
  
ProgramSolverFactory
  
ProgramSolverStatus
  
*ProgressMonitor*
  
*QuadraticExpr*
  
*QuadraticExpr.Term*
  
*QuadraticProgramSolver*
  
R
  
RandomConstraintsGeneratorV1
  
RandomConstraintsManager
  
RandomEnvGeneratorV1
  
RandomEnvManager
  
RandomFluxVectorManager
  
RandomObjectiveGenerator
  
RandomObjectiveManager
  
RCorrelator
  
RCorrelator.Result
  
RedundantCycleRemoval
  
*ResultsManager*
  
ReverseRxnFluxIntervalComputer
  
ReverseRxnFluxIntervalManager
  
ReversibleRxn
  
RevRxnFormatV1
  
RevRxnFormatV2
  
RKendall
  
RofoofSource
  
RomiFile
  
RomiFileSource
  
RomoFile
  
RomoFileSource
  
RowOrFbaOptOutputFile
  
RowOrTreeMatrix
  
RPearson
  
RSpearman
  
RxnDeletionComputer
  
RxnDeletionManager
  
RxnDeletionProcess
  
*RxnNameExprFormat*
  
RxnNameExprFormatV1
  
*RxnNameFormat*
  
RxnNameFormatV1
  
*RxnNameListFormat*
  
RxnNameListFormatV1
  
*RxnNameOrExprFormat*
  
RxnNameOrExprFormatV1
  
*RxnNameSetFormat*
  
RxnNameSetFormatV1
  
RxnNameVerifier
  
RxnNamExprSetInputFile
  
RxnRemovalManager
  
SbmlWriter
  
SbmlWriterManager
  
Sbrt
  
*SbrtFile*
  
SbrtGui
  
SenseFormat
  
SerFbaOptOutputFile
  
SerializedFileIterator
  
SerializedInputFile
  
SerializedMapInputFile
  
SerializedOutputFile
  
SfoofSource
  
*ShellProcessManager*
  
SimpleFluxIntervalComputer
  
SimpleFluxIntervalManager
  
*SimpleFormat*
  
SimpleListFormat
  
SimpleRxnFileTranslator
  
SimpleRxnFileTransManager
  
SimpleSetFormat
  
SingleElementUnionManager
  
SingleElementUnionProc
  
SingleElementUnionsV1
  
SingleElementUnionsV2
  
SingleRm
  
SingleVectorConverterManager
  
SinkRxn
  
SlpCycleComputer
  
SlpCycleIdManager
  
SlpExCurrComputerV2
  
*SmdMatrix*
  
SmoFile
  
SmoFileSource
  
SourceRxn
  
*SparseDoubleMatrix*
  
SparseDoubleMatrixWrap
  
SpecialChars
  
StoichVariationManager
  
StoichVariationProcess
  
StrictSingleElementUnionManager
  
StrictSingleElementUnionProc
  
StrictSingleElementUnions
  
StringFormat
  
SvCdHarFluxVectorManager
  
SvCdHarFluxVectorProcess
  
SvInitialPointManager
  
SvInitialPointProcess
  
TabListFormat
  
TabSetFormat
  
TauResult
  
Taxicab
  
TextAreaOutputStream
  
TextFileLineIterator
  
TextInputFile
  
TextOutputFile
  
TextPaneGui
  
TextPaneOutputStream
  
TreeMatrix
  
TrueFalseFormat
  
UnboundedVariableException
  
UniqueCycleIdManager
  
UnmodifiableCollection
  
UnmodifiableIterator
  
UnmodifiableList
  
UnmodifiableMap
  
UnmodifiableSet
  
Util
  
VariableParticipation
  
VariableParticipationManager
  
VectorComparer
  
VectorComparer.Diff
  
VectorComparison
  
VectorComparisonManager
  
VectorIntersection
  
*WarningHandler*
  
WarningHandlerFactory
  
WhitespaceListFormat
  
WhitespaceSetFormat
  
WorkingDirectory
  
WwFluxomeReducer
  
WwFluxomeReducerManager
  
XmlFileFactory
  
YesNoFormat
